# The epigenetic regulators and metabolic changes in ferroptosis-associated cancer progression

**DOI:** 10.1186/s12943-020-01157-x

**Published:** 2020-02-27

**Authors:** Yuqing Wu, Siwei Zhang, Xiaoxiao Gong, Samantha Tam, Desheng Xiao, Shuang Liu, Yongguang Tao

**Affiliations:** 1 Department of Pathology, Key Laboratory of Carcinogenesis and Cancer Invasion, Ministry of Education, Xiangya Hospital, Central South University, Changsha, 410078 Hunan China; 2 NHC Key Laboratory of Carcinogenesis (Central South University), Cancer Research Institute and School of Basic Medicine, Central South University, Changsha, 410078 Hunan China; 3 Department of Biomedical and Molecular Sciences, Queen’s University, Kingston, Ontario Canada; 4 Department of Oncology, Institute of Medical Sciences, National Clinical Research Center for Geriatric Disorders, Xiangya Hospital, Central South University, Changsha, 410008 Hunan China; 5 Department of Thoracic Surgery, Hunan Key Laboratory of Early Diagnosis and Precision Therapy in Lung Cancer, Second Xiangya Hospital, Central South University, Changsha, 410011 China

**Keywords:** Ferroptosis, Epigenetics, Cancer, Organelles, Chromatin remodeling factor, lncRNA, Golgi, Lysosome, Endoplasmic reticulum, Mitochondria, Metabolism, Iron, Lipid peroxidation, Immunotherapy

## Abstract

Ferroptosis, a novel form of regulated cell death, is different from other types of cell death in morphology, genetics and biochemistry. Increasing evidence indicates that ferroptosis has significant implications on cell death linked to cardiomyopathy, tumorigenesis, and cerebral hemorrhage to name a few. Here we summarize current literature on ferroptosis, including organelle dysfunction, signaling transduction pathways, metabolic reprogramming and epigenetic regulators in cancer progression. With regard to organelles, mitochondria-induced cysteine starvation, endoplasmic reticulum-related oxidative stress, lysosome dysfunction and golgi stress-related lipid peroxidation all contribute to induction of ferroptosis. Understanding the underlying mechanism in ferroptosis could provide insight into the treatment of various intractable diseases including cancers.

## Background

Epigenetics, including DNA methylation, histone modifications, chromatin remodeling and noncoding RNAs such as long non-coding RNAs (lncRNAs) are important mechanisms in a cell’s adaptability to a number of signals, conditions, and stressors [1]. The reprogramming of gene expression contributes to tumorigenesis and cancer progression. The gene regulatory machinery and its chromatin-associated factors integrate environmental signals to modulate homeostatic responses. Numerous interplays between inter-metabolites and chromatin modification factors have recently been addressed [2]. There is a significant alteration in cellular metabolism, which is believed to associate with chromatin-remodeling events in different cancers [1–3]. These emerging viewpoints have biological relevance to cell fate such as cell death, disease and cancer.

Ferroptosis, a recently defined type of regulated cell death (RCD), is different from other types of cell death in morphology, genetics and biochemistry. A great variety of human diseases including cancer have been linked to the abnormal function of ferroptosis, either in excessive induction or inhibition of it [4–6]. The relationship between ferroptosis and oncogenic Ras has been identified [7]. Ras contributes to the induction of ferroptosis through the RAS-BRAF (B-Raf proto-oncogene, serine/threonine kinase)-MAP2K/MEK (mitogen-activated protein kinase kinase)-MAPK/ERK (mitogen-activated protein kinase) pathway [8]. Also, the role of tumor suppressor gene, tumor protein p53 (TP53) has been revealed in ferroptosis. TP53 represses the cystine/glutamate transporter, solute carrier family 7 membrane 11 (SLC7A11), which regulates the expression of glutathione (GSH), therefore promoting ferroptosis [7, 9]. It is interesting to understand the interplay of epigenetic modifications and metabolic pathways in ferroptosis and tumorigenesis.

Among various differences between normal cells and cancer cells, one distinct characteristic of cancer cells is the switch to an anaerobic metabolism even in the presence of oxygen, known as the Warburg effect. Observations in various cancers have indicated that there is a change of cellular metabolism with the significant changes of the tricarboxylic acid (TCA) cycle, a critical oxidative circulation in metabolism [10–12]. Iron, a critical metallic element, is necessary for cell replication, metabolism and growth [13]. The Fenton reaction is an important part of ferroptosis, in which the lipid peroxidation is mediated by carbon and oxygen centred radicals, initiated by free intracellular iron. Ferrous iron donates an electron in a reaction with hydrogen peroxide to produce the hydroxyl radical, a reactive oxygen species (ROS). This reaction not only damages lipids and proteins, but also causes oxidative damage to DNA [14]. The release of free iron catalyzed by heme oxygenase (Hmox1) generates ROS in the mitochondrial membrane and leads to ferroptosis against doxorubicin (DOX)-treated cardiomyopathy [4], indicating that inference of ferroptosis is feasible as a novel strategy for the treatment of diseases.

Epigenetic regulators determine the gene transcription, cellular fate, developmental processes, and immune cell development of an organism [15]. A panel of epigenetic regulators have recently been linked to the induction of ferroptosis and oncogenesis. These provide a more comprehensive and more exact mechanism in ferroptosis-targeted tumor intervention. And the epigenetic controls in ferroptosis present a new direction for therapeutic intervention, which may help to overcome the current barrier in anti-cancer therapy. Here we discuss various epigenetic regulators and metabolic changes in respect to ferroptosis-mediated cancer cell progression.

### The discovery of ferroptosis and the location of ferroptosis

#### The discovery of ferroptosis

Ferroptosis is a form of RCD driven by iron-dependent lipid peroxidation. It was initially caught when seeking small molecular compounds for targeting RAS mutations, an oncogene. In 2003, a small molecule compound termed ‘erastin’ was identified, which selectively induced nonapoptotic cell death in both an ST- and RASG12V-dependent manner [16]. In 2007, tests of the genotype-selective antitumor activity of erastin in various RAS mutation cancer cell lines confirmed that the RAS-BRAF-MAP2K/MEK -MAPK/ERK pathway and VDAC (voltage-dependent anion channel), mediating oxidative stress and mitochondria dysfunction, respectively, were required for erastin-induced cell killing [17]. VDAC2 and VDAC3 can also be directly targeted by erastin [17]. In 2012, the erastin-induced cell death was finally defined as an iron-dependent RCD and was named ‘ferroptosis’ [7]. Erastin can inhibit the cystine/glutamate transporter system x_c_^−^, leading to cysteine starvation, GSH depletion, and consequently, oxidative death [7]. The discovery of ferroptotic cell death provides insight into cancer research.

Two main components contribute to ferroptotic cell death: increased free iron and accumulated lipid peroxides [7]. Erastin-induced iron accumulation, which can be avoided by iron chelation, antioxidants or the genetic inhibition of cellular iron uptake, is conductive to ROS production, which results in lipid peroxidation and subsequent death [7]. Free iron changes in the labile iron pool (LIP) occurs as a result of increased uptake, decreased storage, breakdown of iron-containing proteins, or malfunction of iron exporters. Almost all intracellular iron can be found in heme-containing and mitochondrial proteins, in the form of iron-sulfur clusters or stored as ferric iron (Fe^3+^) by ferritin. Ferrous iron can react chemically with hydrogen peroxide to form hydroxyl radicals, which lead to an excessive accumulation of free iron after reacting with polyunsaturated fatty acid (PUFAs) to form lipid peroxides. Degradation of ferritin occurs via ferritinophagy, a form of autophagic degradation of ferritin by nuclear receptor coactivator 4 (*NCOA4*) [18, 19]. Accordingly, genetic knockdown or overexpression of *NCOA4* has been shown to prevent or trigger erastin-induced ferroptosis respectively. Taken together, meticulous regulation of heme, the mitochondria, and ferritin are all critical mediators of the LIP, lipid peroxide formation and subsequent ferroptosis.

Ferroptosis is evidently different from apoptosis. It is not effective to block ferroptosis with inhibitors of caspase, cathepsin or calpain proteases, RIPK1 (receptor-interacting serine/threonine kinase 1), PPID/cyclophilin D, lysosomal function or autophagy or the genetic inhibition of apoptosis effectors [7]. However, to some extent, ferroptosis is a type of autophagy-dependent cell death (ADCD) in some cancer cells, resulting from tumor heterogeneity or potential drug stability. Further studies argue a crosstalk between ferroptosis and other established cell death modalities including apoptosis and necroptosis. For example, fibroblasts that are MLKL (mixed lineage kinase domain like pseudokinase)-deficient and necroptosis-resistant are more vulnerable to erastin-induced ferroptosis [20]. Erastin can also induce apoptosis in lung (e.g. A549) and colorectal cancer cell lines (e.g., HT-29, DLD-1, and Caco-2) via the activation of TP53 and mitochondrial oxidative injury [21, 22]. More recently, the major pro-ferroptosis activity of erastin has been linked to directly blocking system x_c_^−^. These findings raise questions about the theoretical foundations of ferroptosis, and the interplay between ferroptosis and apoptosis.

#### The mitochondrion is a crucial player in ferroptosis induced by cysteine deprivation

Mitochondria are vital organelles involved in energy metabolism, cell signaling, and cell death pathway regulation including ferroptosis. Moreover, ferroptosis is morphologically depicted by condensation of mitochondria, reduction of mitochondrial cristae, and decrease in mitochondrial size [23–25]. It appears reasonable that the mitochondrion is indeed a crucial player in ferroptosis induced by cysteine deprivation. However, it remains highly controversial whether or not mitochondria are an important component in ferroptosis. Ferrostatin-1 is a potent and selective inhibitor of ferroptosis, which accumulates in specific organelles including lysosomes, mitochondria, and endoplasmic reticulum (ER), but not the plasma membrane or nucleus. Nevertheless, mitochondria are not indispensable for ferroptosis or ferroptosis rescue by Ferrostatin-1 [26]. Further work is needed to excavate certain statuses of mitochondria in ferroptosis.

Mitochondria play a vital role in ferroptosis induced by cysteine deprivation but not by glutathione peroxidase-4 (GPX4) inhibition, the most downstream of the ferroptosis pathway [27]. Cysteine deprivation leads to mitochondrial membrane hyperpolarization and lipid peroxide accumulation. Inhibition of the mitochondrial TCA cycle or electron transport chain (ETC) mitigates mitochondrial membrane hyperpolarization, lipid peroxide accumulation, and ferroptosis [27]. Glutaminolysis (a major source of anaplerosis) is involved in ferroptosis through ferroptotic functioning of the TCA cycle. Importantly, loss of fumarate hydrase function, a TCA cycle component and tumor suppressor, confers resistance to cysteine-deprivation induced ferroptosis [27]. Therefore, both mitochondrial TCA cycle and the ETC action are dispensable to potent ferroptosis.

#### The endoplasmic reticulum (ER) is involved in ferroptosis

Containing more than half of all lipid bilayers in any cell, ER is the source of lipids for most membranes in other organelles and may also be critical to ferroptosis initiation. The ER lumen is an oxidative environment and may initiate ferroptosis as a consequence of oxidative stress [26]. System x_c_^−^ is an antiporter that imports one molecule of cystine in exchange for one molecule of glutamate. Small molecule inhibition of system x_c_^−^, such as erastin and its analogs, specifically inhibit cystine uptake via system x_c_^−^, which leads to ER stress,triggering ferroptosis in a variety of cellular contexts. Upregulation of ER stress markers such as *CHAC1* (ChaC, cation transport regulator homolog 1), ATF4 (activating transcription factor 4) and phosphorylation of eIF2α have been observed in ferroptosis [28]. Ferroptotic agents erastin and artesunate (ART) can induce ER stress and promote p53 upregulated modulator of apoptosis (PUMA) expression via C/EBP-homologous protein (CHOP), whereas ER stress response mediated by the PERK (PKR-like ER kinase)-eIF2α (eukaryotic initiation factor 2α)-ATF4 pathway is involved in regulation of the expression of several target genes such as *CHOP* [29–31]. ATF4 is also involved in upregulation of the heat shock 70 kDa protein 5 (HSPA5, also termed GRP78 or BIP), a member of the molecular chaperones expressed primarily in the ER, resulting in the inhibition of lipid peroxidation in ferroptosis by directly protecting against GPX4 degradation [32]. These ER-related regulators significantly contribute to ferroptosis.

**Fig. 1:**
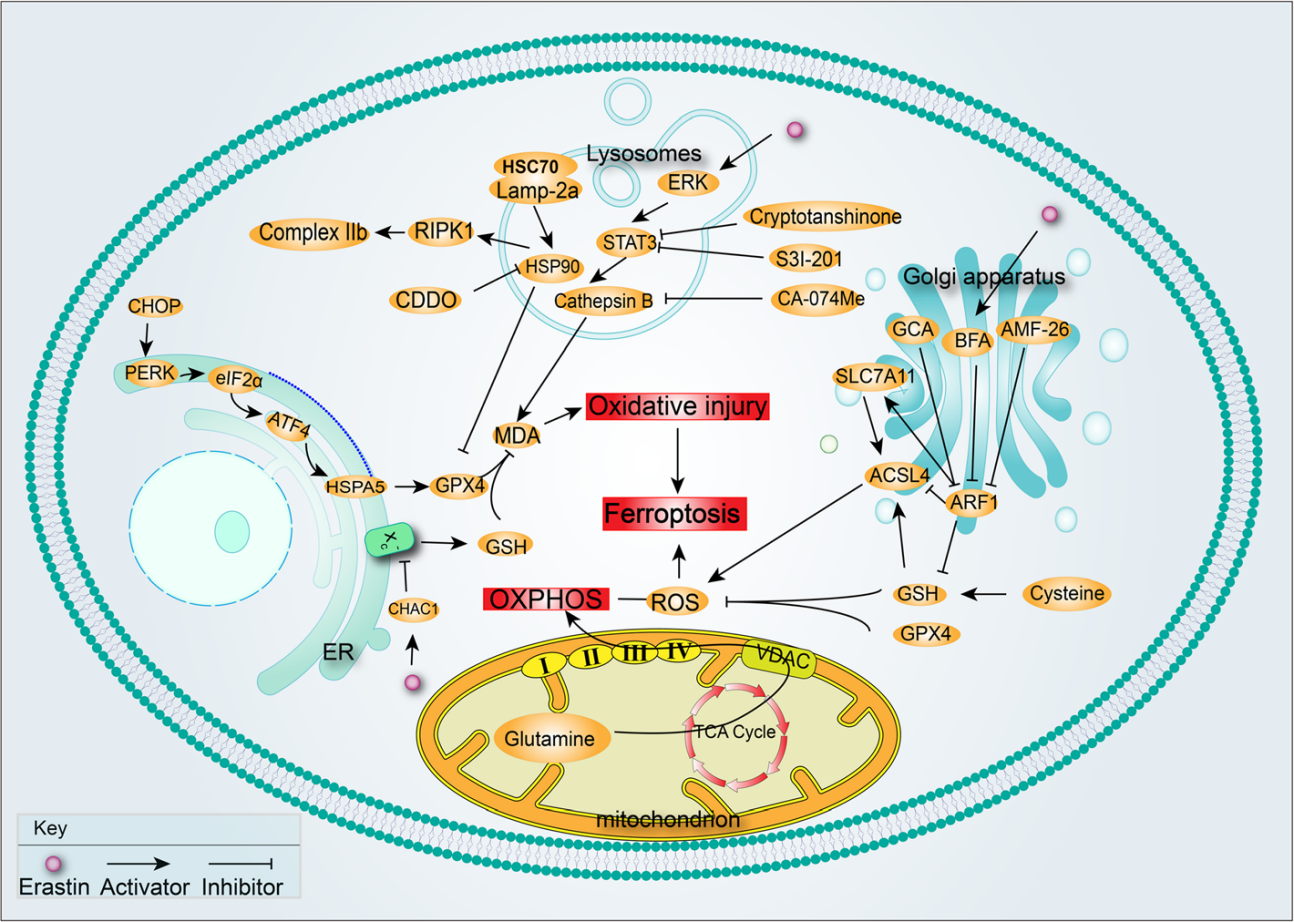
Known function of organelles in ferroptosis. In the mitochondrion, glutamine is oxidatively phosphorylated, contributing to ROS production. The ER stress response is mediated by the PERK-eIF2α-ATF4 pathway involved in GPX4 degradation, ultimately, inhibiting oxidative injury. In the lysosome, STAT3-mediated cathepsin B expression is required for ferroptosis via the MEK-ERK pathway. Meanwhile, chaperone-mediated autophagy (CMA) regulated by HSP90, CDDO, HSC70 and Lamp-2a promotes the degradation of GPX4. In the Golgi apparatus, Golgi-disrupting compounds enable inhibition of ARF1, an inhibitor of GSH and ACSL4, and activator of SLC7A11, leading to increased ROS

In addition, ER also participates in ferroptosis rescue. The hypothesis that the location in which ferrostatin exerts its antiferroptotic action is the ER, is supported by the increased potency of ferrostatin-1 in postmitophagy cells, which shows greater ER abundance [26]. Relevant regulators and pathways have been discussed, while the link of ferroptosis and the ER requires further identification (Fig. 1).

#### Ferroptosis is a lysosomal cell death process

Lysosomes contain hydrolases (e.g. the cathepsin family) for the degradation and recycling of essential nutrients to maintain homeostasis through various ways, including ferroptosis [33]. Increasing evidence has demonstrated that lysosomal activity is involved in the induction of ferroptosis [34–38]. Interestingly, ferroptosis as a lysosomal cell death process requires activation of signal transducer and activator of transcription 3 (STAT3)-mediated cathepsin B (mediator in lysosomal cell death) expression [34]. STAT3 promotes erastin-induced ferroptosis through activation of lysosomal cell death in human pancreatic ductal adenocarcinoma (PDAC) cell lines [34]. Meanwhile, in PDAC, erastin promotes, and sorafenib inhibits STAT3 phosphorylation, triggering increased expression of cathepsin B via the MEK-ERK pathway [38]. The process above renders more MDA (malondialdehyde), one of the final products of lipid peroxidation [38]. Taken overall, STAT3-meidated cathepsin B expression is required for ferroptosis with increased oxidative injury in PDAC cells through induction of lysosomal cell death.

Another pathway occurring in lysosomes takes part in ferroptosis as well. Chaperone-mediated autophagy (CMA), a cellular lysosome-mediated degradative mechanism, is involved in the execution of ferroptosis [38]. The activation of ferroptosis increases the levels of lysosome-associated membrane protein 2a (Lamp-2a) known to be associated with heat shock cognate 70 (HSC70) to promote CMA and the degradation of GPX4. HSP90 associates with Lamp-2a at the lysosomal membrane and regulates the functional dynamics of the Lamp-2a complexes for CMA activation. Accordingly, 2-amino-5-chloro-N,3-dimethylbenzamide (CDDO), as a potent inhibitor of ferroptosis, can inhibit HSP90 to block GPX4 degradation, lipid peroxidation, ROS accumulation and cell death consequently [39]. Interestingly, only lysosome pathway inhibitors could inhibit degradation of GPX4 induced by glutamate or erastin rather than proteasome inhibitors, suggesting that degradation of GPX4 might be located in the lysosome [38]. Noticeably, HSP90 is important for mediating the activation of RIPK1 kinase in both necroptosis and RIPK1-mediated apoptosis (RDA), which suggests that HSP90 may represent a common regulatory nodal between necroptosis and ferroptosis. Collectively, CMA regulated by HSP90, CDDO, HSC70 and Lamp-2a promotes the degradation of GPX4 and is ultimately involved in ferroptosis (Fig. 1).

#### Golgi stress involved in ferroptosis

Golgi stress plays a role in ferroptosis in human cells [40]. Ferroptosis can be triggered by several Golgi-disrupting compounds such as AMF-26, BFA, GCA, and AG1478/tyrphostin. Accordingly, diverse ferroptosis-modulating compounds have protective effects on Golgi morphology and functionality after treatment with the above Golgi stressors. Inhibitors of ferroptosis protect cells from Golgi dispersal and inhibit protein secretion in response to several Golgi stress agents. Erastin at sublethal concentrations to cells is sufficient to alleviate Golgi stress-induced lipid peroxidation. The transsulfuration pathway may play a compensatory role for cysteine provision following oxidative challenge as a mechanism to limit ferroptosis [41, 42]. When concurrently applied to cells with a pharmacological inhibitor of the transsulfuration pathway, the pro-survival effects of a low dose of erastin in combination with Golgi stressors can be abolished. The Golgi apparatus is involved in cellular redox control and prevents ferroptosis (.

#### Effects of signal transduction pathways on ferroptosis

There are two central biochemical events, intracellular iron accumulation and lipid peroxidation, leading to ferroptosis. An integrated signaling network plays a vital role in ferroptosis by mediating intracellular iron accumulation, lipid peroxidation and ROS generated by extracellular or intracellular stimuli [43, 44].

#### Iron-mediated oxidative stress

Iron is a trace mineral essential for the functioning of the human body. There are two types of iron existing in the body: heme iron and non-heme iron. Ferric iron (Fe^3+^), the main nonheme iron, can be absorbed by intestinal epithelial cells in the duodenum and upper jejunum, which then binds to transferrin (TF). Fe^3+^ circulates with TF and is then imported into cells through the membrane protein transferrin receptor (TFRC), subsequently reduced to Fe^2+^ in endosomes. Next, Fe^2+^ is released from the endosome into a LIP in the cytoplasm via SLC11A2/DMT1 (solute carrier family 11 member 2/ cytosine-C5 specific DNA methyltransferase). Excess iron can be stored in ferritin or exported into the circulation in the blood stream through the iron efflux pump SLC11A3/ferroportin (solute carrier family 11 member 3). Iron absorption and metabolism have been targeted in the treatment of cancers, while excessive iron can cause tissue injury and increase the risk of developing cancers [13, 45]. The Fenton reaction, catalyzed by iron and responsible for iron biotoxicity, can produce hydroxyl radicals that damage cellular proteins, lipids, and DNA [46]. For example, iron-based nanoparticles can release ferrous and ferric iron in acidic lysosomes, inducing ferroptosis, ultimately suppressing tumor growth [14]. It is evident that iron performs not only physiological but also pathological functions.

Iron uptake, export, utilization, and storage are reprogrammed by ferroptosis [47]. The intracellular levels of Fe^3+^ are upregulated in response to ferroptosis activators [7]. Moreover, ferroptosis can be inhibited by preventing cellular iron overload by knockdown of *TFRC*, inhibiting mitochondrial iron accumulation by the upregulation of the mitochondrial iron exporter CISD1 (CDGSH iron sulfur domain 1) and increasing the storage of iron via the upregulation of cytosolic and mitochondrial ferritin [35, 48–51]. The suppression of IREB2 (iron responsive element binding protein 2), a transcription factor, limits ferroptotic cancer cell death by regulating iron metabolism [7]. In contrast, blockade of iron export by knockdown of *SLC11A3* promotes erastin-induced ferroptosis in neuroblastoma cells [52]. Iron metabolism affects ferroptosis by mediating oxidative stress, reflecting the physiological minitrim of the ferroptotic response.

#### Lipid peroxidation-mediated cytotoxicity

The phospholipid bilayer is the fundamental structure of the biomembrane. Lipids accomplish a diverse range of functions from energy storage to signaling [53]. Lipid peroxidation reflects oxidative damage of biomembranes, lipoproteins, and other molecules containing lipids, which can be implicated in several diseases or pathological conditions, such as atherosclerosis, ischemia-reperfusion injury, heart failure, neurodegenerative diseases, as well as cancer [53–57]. The polyunsaturated fatty-acid-containing phospholipids (PUFA-PLs) is arguably implicated in ferroptosis via lipid peroxidation [58]. Oxidized PUFA-PLs create lipid hydroperoxides during ferroptosis by both enzymatic [the activation of the lipoxygenase-dependent enzymatic pathway involving ACSL4 (acyl-coA synthetase long-chain family member 4) and LPCAT3 (lysophosphatidylcholine acyltransferase 3)] and non-enzymatic (non-enzymatic free-radical chain reactions) pathways [58–61]. As a family of lipid peroxidizing enzymes, lipoxygenases participate in various types of cell death [62–64]. Several lipoxygenases are involved in promoting ferroptosis in human cells, such as ALOX5 (arachidonate 5-lipoxygenase), ALOX12 (arachidonate 12-lipoxygenase, 12S type), ALOX12B (arachidonate 12-lipoxygenase, 12R type), ALOX15 (arachidonate 15-lipoxygenase), ALOX15B (arachidonate 15-lipoxygenase, type B) and ALOXE3 (arachidonate lipoxygenase 3) [58]. Evidently, lipid peroxidation pathways in ferroptotic cancer cell death provide a new target for cancer treatment.

Antioxidant events also play a pivotal role in ferroptosis. Antioxidant defenses counteracting ferroptosis may be divided into four categories: 1) the prevention of Fenton reactions, such as through iron storage [7]; 2) the scavenging or eliminating of free radicals, such as by the production of glutathione (GSH) [7]; 3) the repair of damage from toxic oxidation products, such as by the activation of GPX4 [65]; and 4) adaptive responses, such as upregulation of NFE2L2/NRF2 (nuclear factor, erythroid 2 like 2)-dependent antioxidant protein expression [66] and activation of heat shock response [67]. Overall, it is important to understand the definite relationship between lipid peroxidation pathways and antioxidant systems so as to distinguish ferroptosis from other types of RCD.

#### p53-mediated ferroptosis

p53 is an essential regulator of cell growth, metabolism, differentiation and death. TP53 (tumor protein p53) is famous for its function as a tumor suppressor. In recent years, a mass of research has provided evidence of its pivotal role in metabolic functions, cell apoptosis, growth arrest [68–70], and indicates that p53 plays a bidirectional role in controlling ferroptosis.

p53 can promote ferroptosis to selectively deplete cancer cells via several approaches. Spermidine/spermine N1-acetyltransferase 1 (SAT1), a transcription target of p53, leads to lipid peroxidation and ferroptosis upon ROS stress. In this process, ALOX15 plays a critical role but not GPX4 or SLC7A11 [71]. Through inhibiting the expression of SLC7A11, an acetylation-resistant TP53^3KR^ (K117R, K161R, K162R), can indirectly suppress the absorbency of cystine, which plays a key role in GSH biosynthesis. Moreover, depletion of GSH leads to lipid peroxidation, which then triggers ferroptosis [72]. Furthermore, the loss of K98 acetylation on p53^4KR^ (K98R + K117R + K161R + K162R) compared to p53^3KR^ results in the loss in ability to induce ferroptosis, which indicates that acetylation is pivotal for p53-mediated ferroptosis [73]. p53 targets gene *GLS2* (glutaminases2), relating to glutaminolysis, also involved in ferroptosis [74]. SOCS1 (suppressor of cytokine signaling 1) can sensitize cells to ferroptosis by regulating the expression of some target genes of p53. SOCS1 exerts its function through regulating phosphorylation and stabilization of p53. Interestingly, SLC7A11 and SAT1 are both found as the SOCS1-dependent p53 targets, indicating that the SOCS1-p53 axis is involved in the ferroptosis pathway [75]. On the other hand, p53 also suppresses ferroptosis in other cancer cells (e.g. Colorectal cancer). Loss of p53 prevents accumulation of dipeptidyl-peptidase-4 (DPP4) in the cell nucleus, and contributes to the formation of a complex of DPP4 and NOX1 (NADPH oxidase 1) on the plasma-membrane, thus enhancing lipid peroxidation, which results in ferroptosis. In contrast, by blocking DPP4 activity in a transcription-independent manner, the formation of DPP4-TP53 complex limits erastin-induced ferroptosis [76]. In addition, stabilization of wild-type p53 postpones the onset of ferroptosis and CDKN1A (encoding p21CIP1/WAF1) is required in this process. This delay is also related to slower depletion of intracellular GSH and a reduced accumulation of toxic lipid-ROS [77]. Several other molecules such as lncRNAs, and single-nucleotide polymorphisms can also help p53 play a dual role in ferroptosis [78, 79]. For example, the Pro47Ser polymorphism (S47) can restrain erastin-induced GLS2 expression and ferroptosis [78]. However, the exact mechanisms of the dual functions of p53 in ferroptosis have not been clearly uncovered. The effects of p53 and its epigenetic regulators in ferroptosis may contribute to a potential target in ferroptosis related diseases.

#### NFE2L2/ NRF2- mediated ferroptosis

Many pathological conditions are linked to imbalances in redox homeostasis. It is becoming increasingly apparent that the accumulation of lipid peroxides has an important role in multiple diseases. The transcription factor nuclear factor erythroid 2-related factor 2 (NRF2/ NFE2L2) is a key regulator of the cellular antioxidant response, controlling the expression of genes that counteract oxidative and electrophilic stresses as well as ferroptosis.

In liver cancer cells, ferroptosis activators (e.g., erastin, sorafenib) could enhance the expression of SQSTM1/p62 (sequestosome 1), a competitive inhibitor of KEAP1 (Kelch-like ECH-associated protein 1). The levels of NRF2 are kept basally low by three different E3-ubiquitin ligase complexes: Kelch-like ECH-associated protein 1-Cullin 3-Ring box 1 (KEAP1-CUL3-RBX1), S-phase kinase-associated protein 1-Cullin1-Rbx1/β-transducin repeat-containing protein (SCF/β-TrCP), and synoviolin/Hrd1. Therefore, ferroptosis activators can induce NFE2L2 protein stabilization and its transcriptional activity [66]. SQSTM1 or p62 is a scaffold protein with multiple functions that can be used as a cargo receptor to eliminate intracellular proteins (e.g., KEAP1) through autophagy. Depending on the clearance of autophagic cargos, SQSTM1 plays a dual role in both promoting cell survival and cell death [80]. It is unclear whether there is an interconnection between SQSTM1-mediated autophagy and SQSTM1-NFE2L2-regulated ferroptosis [8]. In addition, the upregulation of metallothionein MT-1G (metallothionein-1G) is strongly associated with sorafenib resistance in human HCC cells, the molecular mechanisms of which involve the inhibition of ferroptosis. Metallothioneins (MTs) are low molecular weight and cysteine-rich proteins that are highly induced in response to different environmental stressors, including metal ions [81]. MT-1G is identified as a downstream target of NFE2L2 that contributes to ferroptosis resistance in response to sorafenib [81]. NRF2 together with MafG (v-maf avian musculoaponeurotic fibrosarcoma oncogene homolog) can activate transcription of NQO1 (quinone oxidoreductase-1), HO1 (heme oxygenase-1), and FTH1 (ferritin heavy chain-1), which have the ability to inhibit ferroptosis. HO1 plays a dual role in the inhibition or promotion of ferroptosis, depending on the cellular redox status. The downregulation of HO1 in renal proximal tubule cells of mice promotes ferroptosis, and high levels of HO1 promotes Bay-induced ferroptosis through NRF2-SLC7A11-HO1 pathway [82]. It is evident that NRF2 participates and plays an essential role in ferroptosis through the different pathways explained.

#### ECAD- induced hippo signaling pathway to inhibit ferroptosis

A group test of human epithelial cancer cells revealed that ferroptosis is dependent on cell density, which increases the interaction between cells. E-cadherin (ECAD) plays an important part in the mediation of intercellular contact in epithelial cells [83, 84]. ECAD expression is positively associated with the level of cell confluence, and promotes intercellular dimerization, therefore, driving the Hippo signaling pathway through mediating NF2. Activation of tumor suppressor NF2 downregulates the expression of an E3 ubiquitin ligase (CRL4-DCAF1), which promotes the oligoubiquitylation of the kinase Warts (LAST1/2) and leads to their degradation [65]. As a result, NF2 reverses the inhibition of kinase activity LAST1/2, which induces the phosphorylation of an oncogenic transcriptional co-activator YAP. This removes YAP from the nucleus, thereby suppressing substrates of YAP, including TFRC1 and acyl-CoA synthetase long chain family member 4 (ACSL4). Collectively, the activation of the cadherin-NF2-Hippo-YAP signaling axis, a pathway dependent on cell density, blocks ferroptosis as a result [85]. Paradoxically, lymphocyte-specific helicase (LSH) inhibits ferroptosis, and downregulates the expression of EMT-related genes including, E-cadherin and ZO-1 to promote cancer progression in IKKα-mediated way [86, 87]. In addition, other substrates of YAP also contribute to the induction of ferroptosis, however the co-overexpression of TFRC and ACSL4 did not restore ferroptosis in confluent cells to the level of that in sparse cells [88, 89]. Mutation of the E-cadherin–NF2–Hippo–YAP signaling axis in various cancers could provide new ideas for current treatments in terms of ferroptosis.

### Regulation of metabolic pathways in ferroptosis

#### VDAC sensitize cells to ferroptosis

Erastin exhibits great lethal potential in human tumor cells containing mutations in the oncogenes *HRAS*, *KRAS* or *BRAF* [17]. In response to erastin, the mitochondria changes in morphology, structure, and function [8, 16]. In the mitochondria, erastin acts through VDACs (voltage-dependent anion channels) found in the outer mitochondrial membrane. Depletion of VDAC2 and VDAC3 reduces erastin harm in RAS-mutated cancer cells, indicating that these mitochondrial membrane proteins are important for ferroptosis [17]. However, whether VDAC2 and VDAC3 are indispensable to ferroptosis requires further investigation. Cells are sensitive to ferroptosis activators despite depletion of mitochondrial DNA. Some other compounds inducing mitochondrial ROS seem not to induce ferroptosis. These facts all fail to support the significance of mitochondria in ferroptosis [24]. Nevertheless, ferroptosis induction is associated with mitochondrial dysfunction. In neuronal cells, erastin-induced ferroptosis was accompanied by BID (BH3 interacting domain death agonist) transactivation of the mitochondria, resulting in the loss of mitochondrial membrane potential, enhanced fragmentation, and reduced ATP levels. The knockout of BID by CRISPR/Cas9 helps the mitochondria retain its morphology and function, and mediates neuroprotective effects against ferroptosis [90]. Additionally, the expression of VDAC1 could be decreased by the bromodomain inhibitor (JQ1), and bromodomain-containing protein 4 (BRD4) is indicated to be a regulator of VDAC1 [91], providing a novel connection between VDAC and epigenetic regulation. In sum, the role of VDACs in ferroptosis and the conditions under which ferroptosis is promoted requires further investigation.

#### SLC7A11 (system x_c_^−^) plays a key role in ferroptosis as a common target

System x_c_^−^ is an amino acid antiporter, consisting of the functional subunit, SLC7A11 (solute carrier family 7 member 11), and the regulatory subunit,SLC3A2 (solute carrier family 3 member 2) [92], which imports the cystine into cells with a 1:1 counter-transport of glutamate [93]. The synthesis of GSH, a main endogenous antioxidant, depends on the activity of GCL (glutamate-cysteine ligase). The inhibition of system x_c_^−^ inhibits its activity, thus leading to the depletion of intracellular GSH, contributing to higher levels of lipid peroxide, resulting in ferroptosis [56]. The upregulation of system x_c_^−^ expression may be involved in chemoresistance and tumor growth [94, 95]. In breast cancer cells, in order to respond to ROS, System x_c_^−^ is upregulated to biosynthesize more GSH. The light chain subunit of system x_c_^−^, xCT, is regulated by NRF2 [94]. ARF (ADP-ribosylation factor) can restrain the overexpression of NRF2, whose down targets include SLC7A11. Thus, ARF expression sensitizes cells to ferroptosis [96].

Several studies have revealed the relationship between small molecule and system x_c_^−^. Erastin binds to system L (including SLC7A5/SLC3A2 complex) especially SLC7A5 (solute carrier family 7 member 5), a major part to transport neutral amino acids [24]. p53^3KR^ is an acetylation-defective mutant unable to induce cell-cycle arrest, apoptosis and senescence, however, retaining its ability to inhibit SLC7A11 and indirectly reduce the activity of system x_c_^−^ [72]. The BECN1 (beclin 1) network, an important protein in the regulation of autophagy, also plays a vital role in ferroptosis. AMP-activated protein kinase (AMPK) mediates the phosphorylation of BECN1 at Ser90/93/96, and then BECN1 combines with SLC7A11, blocking its activation [97]. This indicates a potential link between autophagy and ferroptosis. Activation transcription factor 3 (ATF3), a member of the ATF/CREB family of transcription factors, is highly expressed when cells are under stress, including DNA damage and oxidative stress. ATF3 puts cells to a state where cells are sensitive to erastin-induced ferroptosis. This is done by ATF3 through binding to the SCL7A11 promoter at BS-1/BS-2 sites and repressing the expression of SLC7A11 [98]. All abnormal regulations of the molecule above can result in the decrease of cysteine in cells, leading to lipid peroxidation accumulation, resulting in ferroptosis. Various pathways have been found in mediating ferroptosis. Of note, some of the pathways take SCL7A11 as their common target which indicates that SCL7A11 plays a key role in ferroptosis.

#### GPX4 inhibits ferroptosis

GPX4 is a member of GSH peroxidase that can reduce lipid peroxides in cells and help cells survive [99]. Loss of activity of GPX4 promotes ferroptosis [65, 100]. RSL3(RAS-selective lethal 3), a ferroptosis inducer, has a critical role in GPX4- regulated ferroptosis. Unlike erastin that promotes ferroptosis either by downregulating GSH or via the VDAC2/VDAC3 mechanism, RSL3 can covalently bind to a nucleophilic active site “selenocysteine” of GPX4 depending on its electrophilic chloroacetamide and subsequently do harm to the activity of GPX4. FINO2 (endoperoxide-containing 1,2-dioxolane) can also initiate ferroptosis through GPX4 inactivation. FINO2 requires both an endoperoxide and a nearby hydrophilic head to induce ferroptosis. However, the exact relationship between GPX4 and FINO2 remains an enigma [101]. Depletion of GPX4 increases phospholipid hydroperoxide and promotes lipoxygenase-mediated lipid peroxidation, which ultimately leads to ferroptosis [65, 100].

The abnormal expression of GPX4 is shown to link with various human diseases including cancer and chronic disease. GPX4 is higher expressed in cancer tissues than normal and is negatively associated with prognosis of patients, through hypomethylation in the upstream of GPX4, and active epigenetic modifications at H3K4me3 (trimethylation of histone H3 at lysine 4) and H3K27ac (acetylation of histone H3 at lysine 27) in the transcription start site of GPX4, indicating that high level of GPX4 in cancer may resulted from epigenetic regulation. In addition, GPX4 may potentially be involved in translation of protein, mitochondrial respiratory chain complex I assembly, electron transport oxidative phosphorylation, nonalcoholic fatty liver disease, and metabolic pathways [102]. It has been found that GPX4 depletion-induced ferroptosis occurs in chronic obstructive pulmonary disease (COPD) pathogenesis under cigarette smoke exposure [103]. In addition, NCOA4 (nuclear receptor coactivator 4) mediates ferritin degradation and contributes to disorder of iron homeostasis in COPD lungs, which indicates NOCA4 may involve in ferroptosis [103]. However, the direct link between GPX4 and NCOA4 in terms of protein expression levels remains unknown. It is worth noting that GPX4 can be a latent therapeutic target in some hard-to-treat cancers such as clear-cell carcinomas (CCCs) that have an intrinsic vulnerability to GPX4 inhibition-induced ferroptosis. In renal CCCs, the HIF-2α-HILPDA axis contributes to the sensitivity of CCCs to GPX4 inhibition-induced ferroptosis [104]. HIF-2α (hypoxia inducible factor-2α) activates the expression of hypoxia inducible lipid droplet associated protein (HILPDA), which has the ability to selectively enrich PUFA-TAGs/phospholipids over SFA/MUFA (saturated fatty acids/monounsaturated fatty acids)-lipids and repress adipose triglyceride lipase (ATGL) activity, which enriches polyunsaturated lipids in cells, the downstream of GPX4 [104, 105]. These findings provide a new perspective into the treatment of diseases including cancer involving ferroptosis.

#### Both glutamine and cysteine are important in ferroptosis

Glutamine and cystine are two amino acids required for glutathione (GSH) synthesis, which prevents ferroptosis caused by impaired lipid metabolism including attenuated the accumulation of ROS [106]. Clearly, cysteine deprivation leads to ferroptosis [27]. Many molecules are involved in the regulation of this metabolic pathways. Glutamine (Gln) is an intermediate in the detoxification of ammonia and a well-known nutrient used by tumor cells. SLC38A1 (solute carrier family 38 member 1) and SLC1A5 (solute carrier family 1 member 5) are glutamine transporters that mediate transportation of glutamine through biological membranes. L-Gln is one of the most important amino acids in the body, as it is a nitrogen source in the body and plays an essential role in the TCA cycle [107]. The absorption of Gln mainly depends on SLC38A1 and SLC1A5 [108]. MIR137 (microRNA 137) functions as a negative regulator by downregulating SLC1A5 [109], resulting in the dysfunction of glutamine transporter. The decrease of Gln may be closely related to cystine starvation in cells, thus contributing to ferroptosis as mentioned above [74]. Importantly, loss of function of fumarate hydratase, a tumor suppressor and TCA cycle component, confers resistance to cysteine-deprivation-induced ferroptosis [27]. In addition, glutaminases (GLS) turn Gln into glutamate (Glu) [110]. Glu generated by GLS2-mediated glutaminolysis contributes to form the production of α-ketoglutarate, which may induce ferroptosis [110]. Blockage of glutaminolysis had the same inhibitory effect, which was counteracted by supplying downstream TCA cycle intermediates. Glutamine and cystine, two pivotal components which are involved in various metabolic pathways, are also significant to ferroptosis. Many molecules and proteins such as MIR137 and GLS2 can regulate their expression, which indicates that glutamine and cystine may be a latent common pathway for ferroptosis.

#### Peroxidation of polyunsaturated fatty acids are tightly linked to ferroptosis

Lipoxygenases including ALOXE3, ALOX5, ALOX12, ALOX12B, ALOX15 and ALOX15B, are a family of non-heme iron enzymes involved in generating leukotrienes from arachidonic acid (AA). ALOX12 has an essential function in p53-dependent ferroptosis. p53 activates ALOX12 function by transcriptionally repressing the expression of SLC7A11, leading to ALOX12-dependent ferroptosis upon ROS stress [111]. In addition, polyunsaturated fatty acids (PUFEs) are found to be involved in ferroptosis. When lipid peroxides accumulate to a lethal level in cells, ferroptosis occurs. A study showed that erastin-induced ferroptosis was rescued by silencing either ALOX15B or ALOXE3 in BJeLR and HT-1080 cells [58]. This indicates that in several conditions such as GSH deficiency, lipoxygenases are required for ferroptosis [58]. ALOX5-derived metabolites are related to ferroptosis because NAC (N-acetylcysteine) can counteract toxic lipids generated by nuclear ALOX5 and prevent ferroptosis in mice [112]. PUFA oxidation by lipoxygenases is also essential in GPX4 inhibition, which results in ferroptosis [113]. Clearly, lipoxygenases are tightly related to ferroptosis, however, the exact mechanism of lipoxygenases and their metabolites involved in ferroptosis needs further investigation.

#### ACSL4 is linked with ferroptosis

Human ACSL family, including ACSL1, ACSL3, ACSL4, ACSL5, and ACSL6, expressed on the endoplasmic reticulum and mitochondrial outer membrane, can catalyze fatty acids to form acyl-CoAs [114]. ACSL4 has a preference for long polyunsaturated fatty acids, such as AA and AdA (adenosine deaminase) [59]. This proves that only ACSL4 is related to ferroptosis induced by GPX4 inhibitors or erastin [59, 61]. Overexpressing *ACSL4* promotes ferroptosis. On the contrary, knocking down *ACSL4* prevents ferroptosis and inhibits ferroptosis induced by GPX4 depletion [59, 60]. ACSL4 enriches cellular membranes with long polyunsaturated ω6 fatty acids, promoting the production of 5-hydroxyeicosatetraenoic acid (5-HETE) [60], ultimately causing ferroptosis [61]. Interestingly, miRNA-17-92 might protect endothelial cells from ferroptosis through targeting A20(zinc lipoprotein A20, also known as tumor necrosis factor alpha inducible protein 3) that regulates ACSL4 expression directly, indicating that this microRNA protects cells from ferroptosis [115]. In addition, the inhibition of ferroptosis can ameliorate in situ and remote organ injury, in detail, ACSL4 is induced after ischemia and is involved in ischemia/reperfusion injury in the intestine. Transcription factor Sp1 could upregulate ACSL4 expression by directly binding to the ACSL4 regulatory region [116]. To sum up, ACSL4 is another essential way regulating ferroptosis. MicroRNA mediating ACSL4 plays an indispensable role in this pathway, however, the more details in this pathway need more investigation.

#### The participation of NFS1 and ISCs in ferroptosis

Iron-sulfur clusters(ISCs)function as protein co-factors in a variety of enzymes which are sensitive to oxidative damage. NFS1 (cysteine desulfurase) is an enzyme that is capable of gaining sulfur from cysteine to synthesize ISCs [117]. ISCs are found in mitochondria and play a key role in electron transfer. Suppression of NFS1 cooperating with inhibition of cysteine transport can trigger ferroptosis. The lack of ISCs leads to the iron-starvation response, and in combination with the inhibition of GSH biosynthesis, induces ferroptosis [117]. These results show the participation of NFS1 and ISCs in ferroptosis.

**Fig. 2:**
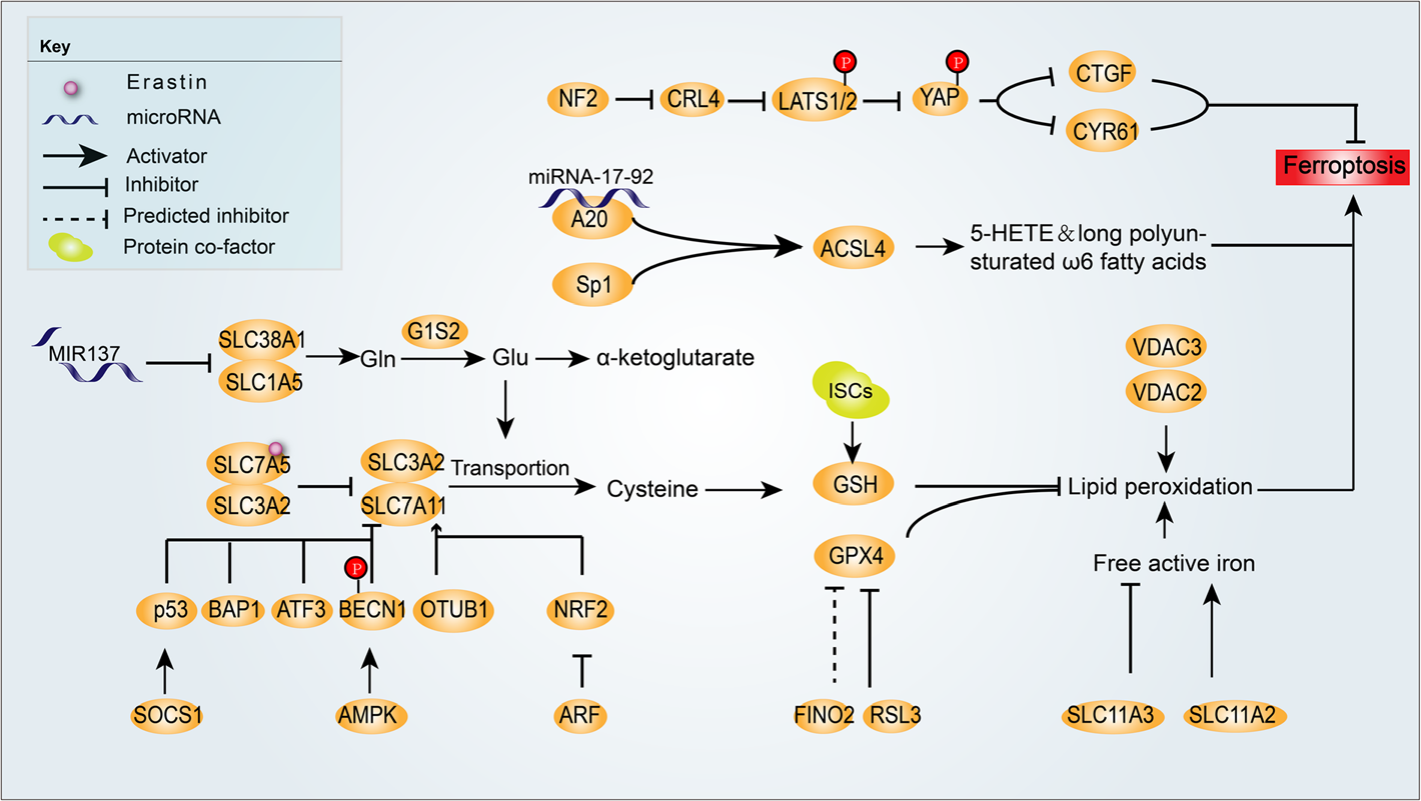
Metabolic pathways in ferroptosis. In the process of ferroptosis, lipid peroxidation plays a key role in triggering ferroptosis. A great number of molecules participate in ferroptosis by regulating the same protein, resulting in similar downstream pathways. For example, p53, BAP1, ATF3, phosphorylated BECN1,and the combination of erastin and SLC7A5, inhibit expression or activation of SLC7A11, which leads to the depletion of cysteine and GSH in cells. Some other molecules such as FINO2 and RSL3 inhibit GPX4 in cells. The decrease of GSH or GPX4 in cells contributes to lipid peroxidation, ultimately resulting in ferroptosis. MIR137 inhibits SLC1A5 and decreases Gln, Glu, and α-KG in cells, also involved in ferroptosis. The cadherin–NF2–Hippo–YAP signaling axis is involved in ferroptosis. miRNA-17-92 prevents ferroptosis in cells through targeting the A20-ACSL4 axis. ACSL4 that is also regulated by Sp1 regulates the expression of 5-HETE and long polyunsaturated ω6 fatty acids in cells, thus participating in the regulation of ferroptosis

To sum up, numerous molecules and metabolic pathways are involved in the regulation of ferroptosis. Lipid peroxidation is their common pathway. Studies on lipid peroxidation induced by cysteine reduction and GPX4 reduction are the most extensive, which can both inhibit GSH from converting to oxidized glutathione. The depletion of cysteine decreases the synthesis of GSH; however, the depletion of GPX4 prevents the chemical reactions GSH involved. System x_c_^−^ promotes the synthesis of GSH by increasing the absorption of cysteine, and molecules such as ATF3 and Glu promote ferroptosis by inhibiting this complex. Unlike cysteine regulated by system x_c_^−^, GPX4 is mediated by other pathways such as HIF-2α-HILPDA axis. In addition, ACSL4 which is involved in A20-ACSL4 axis and can alter the lipid components of cell membranes is another important regulator of ferroptosis. Of note, epigenetic regulation like histone modifications and microRNA-mediated gene silencing also plays a vital role in ferroptosis. All in all, the metabolic regulation of ferroptosis is an intricate network. (Fig. 2).

### The epigenetic regulators in ferroptosis

Epigenetic regulators including DNA methylation, histone modifications, and noncoding RNAs, determine the gene transcription, cellular fate, developmental processes, and immune cell development of an organism [15]. Ferroptosis is a form of non-apoptotic cell death characterized by the iron-dependent overproduction of lipid hydroperoxides. Whether nuclear events participate in the regulation of ferroptosis is largely unknown. Studies in recent years have focused on the epigenetic mechanism of ferroptosis. Here, we list some critical epigenetic effectors in ferroptosis regulation.

#### Lymphocyte-specific helicase (LSH) acts as an epigenetic regulator to inhibit ferroptosis

Lymphocyte-specific helicase (LSH), a member of the ATP-dependent helicase in sucrose nonfermenting 2 (SNF2), plays an important role in normal cell development, metabolism and cancer progression [79, 87, 118–129]. Interestingly, LSH inhibits ferroptosis and promotes lung tumorigenesis by affecting metabolic genes through chromatin modification [119]. LSH contributes to the recruitment of the WD repeat domain 76 (WDR76) to the metabolic gene promoters, including stearoyl-CoA desaturase 1(*SCD1)* and fatty acid desaturases 2 (*FADS2).* The upregulated expression of SCD1 and FADS2 blocks ferroptosis by affecting lipid ROS and iron accumulation, two major events in ferroptosis. Notably, Egl nine homolog (EGLNs) and *c-Myc* participate in this pathway. With the help of EGLNs, an enzyme that contributes to the degradation of HIF-1α, *c-Myc* is recruited to the *LSH* gene promoter, upregulating *LSH* expression [86, 130]. Another group also reports that SCD1, an enzyme that catalyzes the rate-limiting step in monounsaturated fatty acid synthesis, inhibits ferroptosis through increasing CoQ10 (Coenzyme Q-binding protein 10), an endogenous membrane antioxidant whose depletion has been linked to ferroptosis [131].

#### lncRNA acts as an epigenetic regulator to promote ferroptosis

lncRNAs are a group of non-coding RNAs that consist of more than 200 nucleotides but possess low or no protein-coding potential. They can interact directly with DNA, mRNA, or proteins to regulate chromatin modification or structure, transcription, splicing, and translation, resulting in the alteration of a variety of physiological and pathological processes such as cell proliferation, differentiation and RCD [132]. As for RCD, remarkably, it has been found that lncRNAs have a place in the cancer cell progression in ferroptosis. For instance, lncRNAs participate in the development and progression of non-small cell lung cancers (NSCLC) through mediating ferroptosis. RNA sequencing in NSCLC cells showed that *SLC7A11*, a key gene associated with ferroptosis through its role in controlling iron concentrations, can be downregulated by XAV939 (an inhibitor of NSCLC), as the target genes of lncRNAs, and suppress the development of NSCLC via ferroptosis-mediated pathways [133].

The cytosolic lncRNA P53RRA promotes ferroptosis by activating the p53 pathway and affecting transcription of several metabolic genes [79]. P53RRA increases erastin-induced growth inhibition, the intracellular concentrations of iron and lipid ROS in NSCLC cells consistent with its role in ferroptosis. P53RRA regulates p53 target genes in the cytoplasm by displacing p53 from a G3BP1 complex through its interaction with Ras-GTPase-activating protein-binding protein 1 (G3BP1), a signal transduction modulator stimulated by the oncoprotein Ras, which leads to higher p53 retention in the nucleus so as to stimulate ferroptosis. Delicate balances in chromatin modification are involved in the regulation of P53RRA. To summarize, lncRNA P53RRA regulated by chromatin modification inhibits ferroptosis-regulated genes in a p53-dependent manner by interacting with G3BP1.

LINC00336 acts as a crucial inhibitor of ferroptosis in carcinogenesis by decreasing intracellular levels of iron and lipid ROS through interacting with ELAVL1 (ELAV like RNA binding protein 1), which has been recognized as a novel regulator of ferroptosis [129]. Moreover, ELAVL1 increases LINC00336 expression by stabilizing its posttranscriptional modifications. Contrary to P53RRA, LSH promotes the expression of LINC00336 by upregulating ELAVL1 through the p53 signaling pathway in lung cancer. LINC00336 serves as an endogenous sponge of microRNA 6852 (MIR6852), a negative upstream regulator of cystathionine-β-synthase (CBS)-mediated ferroptosis inhibition. These findings indicate that lncRNA is a critical regulator for ferroptosis and may serve as an effective target of NSCLC therapy.

#### Deubiquitinase acts as an epigenetic regulator to promote ferroptosis

BAP1 (BRCA1-associated protein) encodes the deubiquitinase (DUB) in the nucleus, a common inactivated tumor suppressor in different cancer cell lines, including uveal melanoma (UVM), renal cell carcinoma, mesothelioma, and cholangiocarcinoma [134]. Genomic analyses together with robust statistics have revealed that the restoration of BAP1 facilitates the formation of the polycomb repressive deubiquitinase (PR-DUB) complex and inhibits ubiquitinated histone 2A (H2Aub) occupancy on the *SLC7A11* promoter. As a result, the downregulation of *SLC7A11* blocks ferroptosis as it leads to cystine starvation and depletion of GSH; an important precursor for membrane lipid peroxidation and subsequent ferroptosis-induced cell death [80]. The knockdown of *SLC7A11* in UMRC6 cells (a BAP1-deficient renal cancer cell line) marginally affects cancer cell proliferation, suggesting that the BAP1-mediated tumor suppression through regulating *SLC7A11* [80]. BAP1 is a tumor suppressor, coding for the nuclear deubiquitinating (DUB) enzyme, which reduces histone 2A ubiquitination (H2Aub) on chromatin. By removing ubiquitin from H2Aub on the SLC7A11, BAP1 can repress SLC7A11 expression [89]. In addition, a current study has linked BRCA1-associated tumorigenesis to the stability of Nrf2 (NF-E2–related factor 2), which regulates antioxidant signaling by decreasing the ROS level [135]. The interplay between BAP1 and BRCA1 together with their roles as epigenetic regulators could contribute to the understanding of ferroptosis in terms of oxidative stress. In addition, OTUB1 (OTU deubiquitinase, ubiquitin aldehyde binding 1) is an ovarian tumor (OTU) family member deubiquitinase. It can directly interact with SLC7A11 and regulate SLC7A11 stability, in the process of which, CD44 may play an essential role [136].

Monoubiquitination of histone H2B on lysine 120 (H2Bub1) is an epigenetic mark generally associated with transcriptional activation, and H2Bub1 negatively regulates the Warburg effect and tumorigenesis likely through controlling the expression of multiple mitochondrial respiratory genes, which are essential for OXPHOS (oxidative phosphorylation), through interacting with pyruvate kinase M2 (PKM2), the rate-limiting enzyme of glycolysis [137]. Interestingly, H2Bub1 regulates the expression of SLC7A11 and a group of ion-binding genes that function in multiple metabolism-related processes, indicating that H2Bub1 is a novel epigenetic regulator of ferroptosis. Moreover, p53 promotes the nuclear translocation of H2Bub1 deubiquitinase USP7 (ubiquitin-specific protease 7) that interacts with, deubiquitinates, and stabilizes p53, functioning as novel regulator of H2Bub1 [138]. In addition, the p53-USP7- H2Bub1 axis regulates SLC7A11 expression and activity during ferroptosis induction by erastin treatment [138].

In sum, the effects of deubiquitinases on epigenetic mark links a novel epigenetic mechanism for the regulation of ferroptosis.

#### Selenium acts as an epigenetic regulator to block ferroptosis

**Fig. 3:**
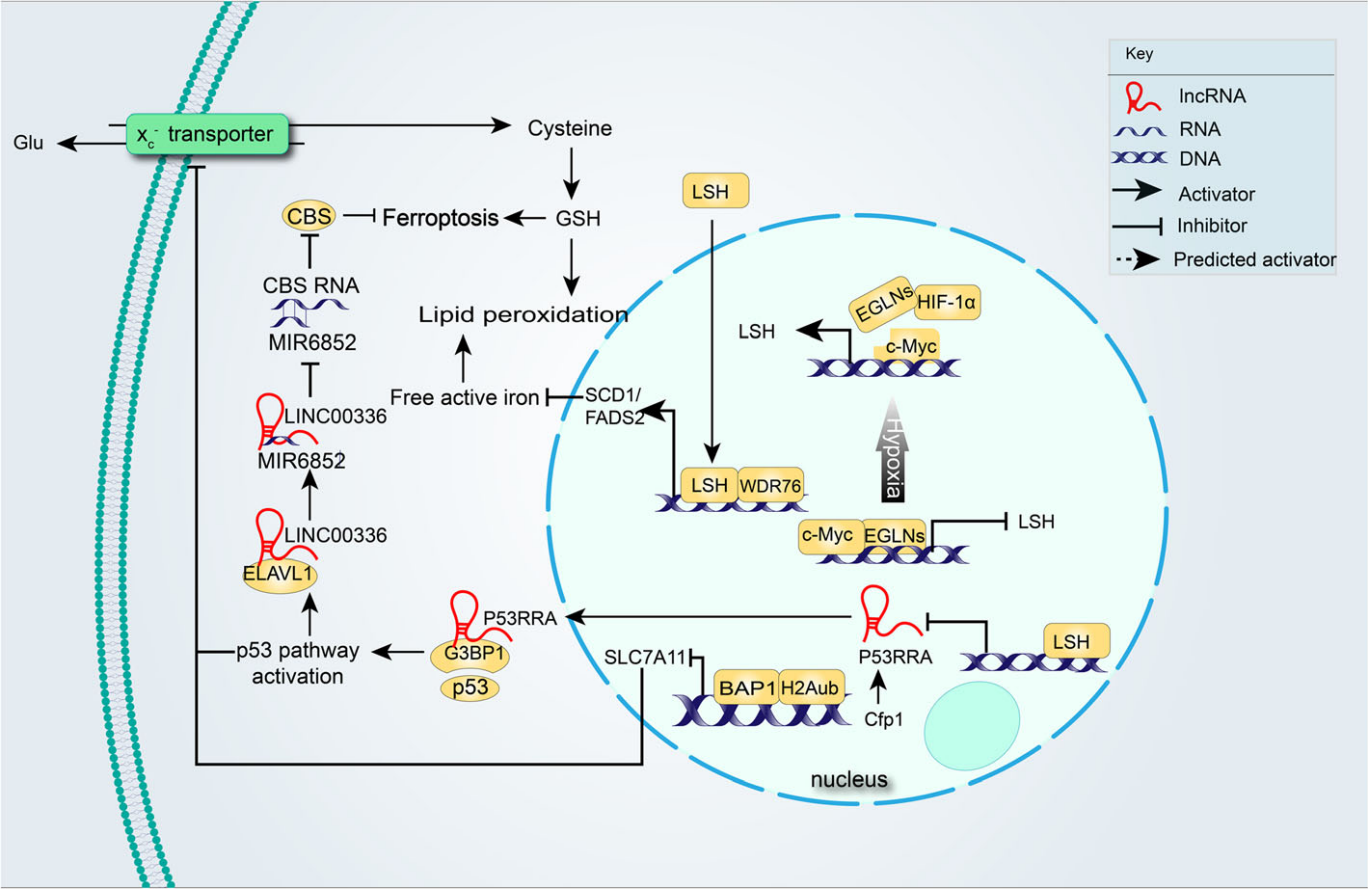
The epigenetic regulators in ferroptosis. The *BAP1*-mediated pathway independent of TP53 executes the suppression of SLC7A11. LSH expression is up-regulated by c-Myc, which is enriched at the LSH promoter by the EGLN1-mediated repression of HIF-1α. The induced LSH interacts with WDR76, which, in turn, up-regulates the lipid metabolic genes including *SCD1* and *FADS2*. LSH also induces ELAVL1 expression through the inactivation of p53 and ELAVL1, enhancing LINC00336 levels. LINC00336 serves as an endogenous sponge of MIR6852 as a circulating extracellular DNA (ceRNA), which increases the mRNA levels of CBS, inhibiting ferroptosis in lung cancer. P53RRA promotes ferroptosis by retaining p53 in the nucleus

Selenium (Se) is an important biosis element and the products of it associate with the health and safety of human beings. Selenoprotein, a type of protein with a selenocysteine (Sec, U, Se-Cys) amino acid residue, includes five antioxidant glutathione peroxidases (GPX) and three thioredoxin reductases (TrxR/TXNRD), which both contain only one Sec. As mentioned above, the expression of GPX4 could counter the induction of ferroptosis; however, the function of its upstream transcription molecules remains unclear [85]. A selenium-mediated way of selenome gene augmentation at the epigenetic level could inhibit ferroptosis and protect neuro cell death after brain hemorrhage [139]. Se recruits TFAP2c (transcription factor activating enhancer-binding protein 2C) and Sp1 to the promoter region of GPX4, upregulating GPX4 expression. The high levels of GPX4 expression are critical for an adaptive response and could reduce cell death after intracerebral hemorrhage by inhibiting ferroptosis. Furthermore, Tat-linked SelP Peptide (Tat SelPep), a selenocysteine-containing peptide that was created artificially, has the same function to improve outcomes after stroke in an Sp1 - dependent manner, providing a wider therapeutic window. Collectively, pharmacological selenium provides insights into a novel therapeutic strategy to block ferroptosis and promote cell survival (Fig. 3).

### The cancer therapeutic application in epigenetic and metabolic regulation of ferroptosis

Protein Kinases have appeared as one of the most intensively interested targets in current pharmacological research, especially for cancer, for their critical roles in cellular signaling transduction. Compared to other kinase inhibitors, sorafenib is the only drug that displays ferroptotic efficacy, through enhancing the expression of SQSTM1/p62, or inhibiting STAT3 phosphorylation, or decreasing the expression level of NRF2 target [38, 81, 140]. In addition, ferroptosis might strengthen the anticancer effect of the apoptosis-inducer cisplatin in cancer cells [141], indicating that ferroptosis inducers could be used to enhance the effect of traditional anticancer drugs. The BRD4 inhibitor (+)-JQ1 (JQ1) has been shown to suppress the proliferation of cancer cells by inducing apoptosis, indicating that JQ1 may be a new therapeutic agent for cancer treatment. (+)-JQ1 could regulate ferroptosis by controlling the expression of ferroptosis-associated genes including GPX4, SLC7A11 and SCL3A2, which regulated by BRD4. (+)-JQ1 regulated ferritinophagy and the expression of ferroptosis-associated genes via epigenetic inhibition of BRD4 by suppressing the expression of the histone methyltransferase G9a or enhancing the expression of the histone deacetylase SIRT1 [142]. Clearly, treatment with JQ1 and RSL3, erastin, or sorafenib produced a satisfactory anticancer effect, suggesting that the combination of JQ1 with ferroptosis inducers could become a new therapeutic modality. A challenge in oncology is to rationally and effectively integrate immunotherapy with traditional modalities including radiotherapy, interestingly, immunotherapy sensitizes tumors to radiotherapy by promoting tumor cell ferroptosis, and IFNγ derived from immunotherapy-activated CD8^+^ T cells and radiotherapy-activated ATM (ataxia telangiectasia mutated) independently, yet synergistically repress SLC7A11 [143], indicating that ferroptosis may be untapped therapeutic mechanism and focus for the development of effective pharmacologic and immunotherapeutic combinatorial approaches with radiotherapy for the treatment of cancer. Moreover, interferon-γ produced by tumor-infiltrating T cells downregulates the expression of SLC3A2 and SLC7A11, two subunits of the glutamate–cystine antiporter system x_c_^−^, impairs the uptake of cystine by tumor cells, and as a consequence, kills cancer cells through the induction of ferroptosis [144], indicating that targeting ferroptosis-associated metabolism in tumors may improve the efficacy of cancer immunotherapy. Interestingly, FePt nanoparticles, a novel ferroptosis agent, could induce ferroptosis by catalyzing the Fenton reaction to produce the ROS. Meanwhile, the metastatic tumors are abolished effectively with the support of oligodeoxynucleotides containing cytosine–guanine together with systemic checkpoint blockade immunotherapy using an anti-CTLA4 (anti-cytotoxic T lymphocyte associated antigen-4) antibody [145], providing a multifunctional platform for anticancer therapeutic applications through ferroptosis. Targeting ferroptosis holds novel promise for the treatment of cancer and considerable efforts are being made to generate ferroptosis modulators for clinical use, but additional studies are required to devise the most efficient strategies in epigenetic and metabolic regulation of ferroptosis.

## Conclusions and perspectives

Intracellular iron concentration and lipid peroxidation are two major biochemical characteristics required for autophagy-dependent ferroptosis, a novel type of RCD. Inhibiting iron overload in the mitochondria and/or blocking the process of redox imbalance can serve as a possible anti-ferroptosis approach [146, 147]. Multiple organelles, including the mitochondria, ER, lysosome, and Golgi apparatus, are involved in the regulation of iron metabolism and lipid peroxidation in ferroptosis. The metabolite-mediated ways of ferroptosis include GPX4 as an important core molecule and system x_c_^−^ in importing cysteine and exporting glutamate. Abundant discoveries have been made in transcription signaling of ferroptosis as well as the effects of different related factors. Among them, NRF2 has been tested to inhibit ferroptosis by targeting system x_c_^−^ and mediating GPX4, therefore, decreasing iron concentration and ROS. However, the targeted effectors of ferroptosis remain unclear and are required for interventions which may contribute to a new way to treat diseases through ferroptosis-induced cell death. Therefore, more detailed research on the suppression of ferroptosis is needed to provide a more valuable therapy.

The mechanistic relationships between epigenetic controls and ferroptosis in cancer progression were previously unclear. However, epigenetic regulators have been identified to involve in ferroptosis and oncogenesis by mediating metabolic genes and intermediators, therefore, contributing to the change of lipid peroxidation. Interestingly, microbial metabolites could not only mediate modulation of host immunity and metabolism as well as epigenetics, but also input the effects on cancer immunotherapy [148], therefore, elevating our understanding of how the microbiome and its metabolites affect cell fate including ferroptosis will advance our capacity to provide well-founded microbial-based therapeutics.

Though the precise and comprehensive role of these factors are not yet fully discovered, they do provide us with a novel way to regulate ferroptosis by mediating potential targets and intervening the axis. The new direction of therapeutic intervention may help to overcome the current barrier in anti-cancer therapy. Further study is required to discover more epigenetic molecules and related mechanisms of ferroptosis, which may contribute to the discovery of anti-cancer therapies.

## Data Availability

Please contact the corresponding author for all data requests.

## Abbreviations

5-HETE: 5-hydroxyeicosatetraenoic acid
A20: zinc lipoprotein A20; tumor necrosis factor alpha inducible protein 3
AA: Arachidonic acid
ACSL: Acyl-CoA synthetase long-chain
AdA: Adenosine deaminase
ADCD: Autophagy-dependent cell death
ALOX: Arachidonate lipoxygenase
AMF-26: A possible novel Arf1-ArfGEF inhibitor
AMPK: AMP-activated protein kinase
ARF: ADP-ribosylation factor
ART: Artesunate
ATF3: Activating transcription factor 3
ATF4: Activating transcription factor 4
ATGL: Adipose triglyceride lipase
ATM: Ataxia telangiectasia mutated
BAP1: BRCA1 associated protein 1
BECN1: Beclin 1
BFA: Brefeldin A
BID: BH3 interacting domain death agonist
BRD4: bromodomain-containing protein 4
CBS: Cystathionine-β-synthase
CDDO: 2-amino-5-chloro-N,3-dimethylbenzamide
CDKN1A: Encoding p21CIP1/WAF1
ceRNA: Circulating extracellular DNA
CHAC1: ChaC, cation transport regulator homolog 1
CHOP: C/EBP-homologous protein
CISD1: CDGSH iron sulfur domain 1
CMA: Chaperone-mediated autophagy
COPD: Chronic obstructive pulmonary disease
CoQ10: Coenzyme Q-binding protein 10
CTLA4: Cytotoxic T lymphocyte associated antigen-4
DMT1: Cytosine-C5 specific DNA methyltransferase
DOX: Doxorubicin
DPP4: Dipeptidyl-peptidase-4
DUB: Deubiquitinating
EGLNs: Egl nine homolog
eIF2α: Eukaryotic initiation factor 2α
ELAVL1: ELAV like RNA binding protein 1
EMT: Epithelial-mesenchymal transition
ER: Endoplasmic reticulum
ERK: Extracellular signal-regulated kinase
ETC: Electron transport chain
FADS2: Fatty acid desaturases 2
FH: Fumarate hydratase
FINO2: Endoperoxide-containing 1,2-dioxolane
FTH1: Ferritin heavy chain-1
G3BP1: Ras-GTPase-activating protein-binding protein 1
GCA: Golgicide A
GCL: Glutamate-cysteine ligase
Gln: Glutamine
GLS: Glutaminases
Glu: Glutamate
GPX4: Glutathione peroxidase-4
GSH: Glutathione
H2Aub: Histone 2A ubiquitination
H3K27ac: Acetylation of histone H3 at lysine 27
H3K4me3: Trimethylation of histone H3 at lysine 4
HIF-1α: Hypoxia-inducible factor α
HIF-2α: Hypoxia inducible factor-2α
HILPDA: Hypoxia inducible lipid droplet associated
Hmox1: Heme oxygenase
HO1: Heme oxygenase-1
HSC70: Heat shock cognate 70
HSP90: Heat shock protein 90
HSPA5: Heatshock 70 kDa protein 5, also termed GRP78 or BIP
IREB2: Iron responsive element binding protein 2
ISCs: Iron-sulfur clusters
KEAP1: Kelch-like ECH-associated protein 1
KEAP1-CUL3-RBX1: Kelch-like ECH-associated protein 1-Cullin 3-Ring box 1
Lamp-2a: Lysosome-associated membrane protein 2a
LIP: Labile iron pool
LMP1: Latent membrane protein 1
lncRNAs: Long non-coding RNAs
LPCAT3: Lysophosphatidylcholine acyltransferase 3
LSH: Lymphocyte-specific helicase
MafG: V-maf avian musculoaponeurotic fibrosarcoma oncogene homolog
MAP2K/MEK: Mitogen-activated protein kinase kinase
MAPK/ERK: Mitogen-activated protein kinase
MDA: Malondialdehyde
MLKL: Mixed lineage kinase domain like pseudokinase
MTs: Metallothioneins
NAC: N-acetylcysteine
NCOA4: Nuclear receptor coactivator 4
NFE2L2: Nuclear factor erythroid 2-related factor 2
NFS1: Cysteine desulfurase
NOX1: NADPH oxidase 1
NPC: Nasopharyngeal carcinoma
NQO1: Quinone oxidoreductase-1
Nrf2: NF-E2–related factor 2
NSCLC: Non-small cell lung cancer
OTUB1: OTU deubiquitinase, ubiquitin aldehyde binding 1
OXPHOS: Oxidative phosphorylation
PERK: PKR-like ER kinase
PR-DUB: Polycomb repressive deubiquitinase
PUFA-PLs: Polyunsaturated fatty-acid-containing phospholipids
RCD: Regulated cell death
RDA: RIPK1-mediated apoptosis
RIPK1: Receptor interacting protein kinase 1
ROS: Reactive oxygen species
RSL3: RAS-selective lethal 3
S47: Pro47Ser polymorphism
SAT1: Spermidine/spermine N1-acetyltransferase 1
SCD1: stearoyl-CoA desaturase 1
SCF/β-TrCP: S-phase kinase-associated protein 1-Cullin1-Rbx1/β-transducin repeat-containing protein
Se: Selenium
Sec, U, Se-Cys: Selenocysteine
SFA/MUFA: Saturated fatty acids/monounsaturated fatty acids
SLC11A2: Solute carrier family 11 member 2
SLC1A5: Solute carrier family 1 member 5
SLC38A1: Solute carrier family 38 member 1
SLC7A11: Solute carrier family 7 membrane 11
SNF2: Sucrose nonfermenting 2
SOCS1: Suppressor of cytokine signaling 1
SQSTM1/p62: Sequestosome 1
STAT3: Signal transducer and activator of transcription 3
TCA: Tricarboxylic acid
TF: Transferrin
TFAP2c: Transcription factor activating enhancer-binding protein 2C
TFRC: Transferrin receptor
TP53: Tumor protein p53
USP7: Ubiquitin-specific protease 7
UVM: Uveal melanoma
VDAC: Voltage-dependent anion channel
WDR76: WD repeat domain 76
α-KG: α-ketoglutarate
